# Quasi‐experimental quantitative study of training programme for nurses and midwives regarding provision of neonatal resuscitation in selected governmental hospital, (Sudan), 2018

**DOI:** 10.1002/nop2.1346

**Published:** 2022-09-08

**Authors:** Namarig Nasraldin Gidam, Widad Ibrahim Abdelgair

**Affiliations:** ^1^ Ministry of Health, Arrass General Hospital Alrass Saudi Arabia; ^2^ Nursing Department, College of Applied Medical Sciences (ALTAIF) University Altaif City Saudi Arabia

**Keywords:** advance, neonatal resuscitation, nurses and midwives, training programme, training programme

## Abstract

**Aims:**

To measure the effectiveness of educational programmes for nurses regarding knowledge and practice of advanced resuscitation for newborn infants.

**Design:**

This is a quasi‐experimental study in (Sudan, White Nile state), to evaluate the effectiveness of designed guidelines regarding advanced neonatal resuscitation for midwives during the period November 2020–January 2021.

**Methods:**

Data were collected using two tools: semi‐structured questionnaire and checklist. The number of the respondents to the questionnaire is 75 nurses. Statistical analyses were conducted using SPSS (version 22). Data were expressed as percentages. The results show that the level of knowledge is improved significantly after training programme interventions, with safe practice reaching (98.70%) compared with a pre‐test percentage of (11.5%). This indicates a steep rise in safe practice after the simulation section, a sharp decrease in unsafe practice after the practical section, followed by subsequent decrease in neonatal mortality rate. This paper has been guided by (STROBE, cohort study) checklist.

**Results:**

The study concluded that the majority of nurses and midwives have some knowledge regarding advanced neonatal resuscitation but still, there is a practice gap because of a shortage of facilities and lack of training, However, training programmes do add value on knowledge and practice for nurses and eventually decrease neonatal mortality rate.

## 1 INTRODUCTION

Nurses and midwives constitute the largest group of healthcare professionals globally (Basu, 2014). Their credentials and experience impact their ability to respond to healthcare crises.

The study was conducted at White Nile State, Kosti and Rabak locality, which lies in west of Khartoum state. Although birth complications are considered critical clinical problems, they may not cause immediate death, unless there is a delay in assisting the non‐breathing newborn to establish adequate ventilation. Such delays may exacerbate hypoxia, increase the need for assisted ventilation, lead to long‐term neurological disability and/or contribute to newborn morbidity and mortality.

Wall et al. (2009). The shortage of nurses combined with political instability and human‐made disasters in the Eastern Mediterranean Region have hindered their development. (Khudhair, 2011; Sintayehu et al., 2020). Boosting the critical role of these professions and alleviating the challenges they face are recognized by WHO as major strategic goals (Basu, 2014).

A recent review showed that the leading causes of mortality and disability of neonates in Arab countries which are lack of neonatal asphyxia prevention and management have directly impacted the nursing and midwifery professions. WHO has consistently raised concerns about infant morbidity and mortality. One of the key millennium development goals was to reduce childhood mortality by 2015. Although the presence of skilled midwives at birth is a key component to improving life chances of newborns with asphyxia, this component of care remains a challenge that defies implementation in developing countries. Inaction to implement newborn resuscitation education and competency assessment remains problematic due to limited availability of equipment, and a lack of standardized continuing professional education, skill development programmes and protocols. In addition, a lack of resources and training is a major contributor also to ineffective newborn resuscitation in some developing countries. Midwives have a crucial role in improving newborn outcomes through appropriate knowledge and skilled resuscitation, but little is known about midwives' resuscitation competence and knowledge.

## 2 BACKGROUND

Lack of knowledge and performance of neonatal resuscitation does not only threaten the lives of the babies, but also affects their mothers, although it can be avoided by taking the necessary precautions and by adequate education. Nurses and midwives represent the cornerstone in maintaining and saving the lives of neonates.

### 2.1 Statement of the problem

Globally, 2.6 million newborn died in 2016 (7,000 deaths every day) (Khudhair, 2011; Sintayehu et al., 2020). The vast majority of deaths took place in developing countries which accounted for approximately 70% of the total. About 39% of the deaths occurred in South‐east Asia followed by sub‐Saharan Africa (SSA) (38%). Five countries from low‐ and middle‐income countries (LMICs), namely India, Pakistan, Nigeria, Democratic Republic of Congo and Ethiopia, accounted for half of all the global newborn death (Shikuku et al., 2017; Khudhair, 2011). Sub‐Saharan Africa (SSA) is a region that suffered from some of the highest mortality rates in the world with one child in 13 dying on the first birthday compared with one in 189 in high‐income countries (Khudhair, 2011; Sintayehu et al., 2020). Five SSA countries, Nigeria, DR Congo, Ethiopia, Tanzania and Uganda, accounted for 50% of the total African newborn deaths (Malekzadeh et al., 2015). Neonatal mortality rate in Ethiopia had remained high (29 of 1,000), contributing by 120,000 deaths every year. (Kim et al., 2013; Suresh et al., 2017). Asphyxia is one of the leading causes of neonatal mortality and morbidity globally (Biset, 2018; Murila et al., 2012; Sintayehu et al., 2020). According to the WHO report of 2012, about one‐quarter of the global neonatal deaths are caused by asphyxia. Furthermore, the WHO 2015 report indicates that it is responsible for 23% of neonatal mortality and 11% of all under five mortalities. (Ezenduka et al., 2016; Wall et al., 2009). Furthermore, and according to the 2013 report of Global Development Alliance (GDA), of 139 million newborns, 16 million babies needed help to breathe with simple resuscitation, 1 million babies needed help to breathe with advanced resuscitation and close to 700,000 died from asphyxia (Mahaling, 2015). This figure had suggested that asphyxia had an overwhelming effect on neonatal survival, and therefore, healthcare professionals (HCPs) have since provided assistance services to enhance the proficiency of resuscitation technique to avert asphyxia‐related morbidities and mortalities.

The burden of asphyxia is disproportionately concentrated in low‐ and middle‐income countries (LMICs) with the highest incidence occurring in sub‐Saharan Africa (SSA) countries (Malekzadeh et al., 2015). It is apparently responsible for 27%–30% of neonatal deaths in resource‐limited countries (Gebreegziabher et al., 2014). Asphyxia is the second most common cause of neonatal mortality in SSA accounting for 24% of neonatal mortality (Malekzadeh et al., 2015). Studies in Nigeria and Zambia had indicated that asphyxia had contributed the highest proportion of neonatal mortality (Ashish et al., 2017; Biset, 2018). According to Ethiopia Demographic and Health Survey (EDHS) 2011, asphyxia is the second most common cause of neonatal mortality in Ethiopia (25%). A study in southwestern Ethiopia had indicated that 47.5% of neonatal mortality is attributed to asphyxia. Furthermore, a study in northern Ethiopia revealed that asphyxia had contributed 31% of neonatal mortality (Callaghan‐Koru et al., 2013; Sintayehu et al., 2020).

Asphyxia is not only the cause for neonatal mortality, but also the cause of a serious long‐lasting morbidity among survivals. Half of asphyxia survivals in developing countries suffered from long‐term abnormal neurological cases. (Ashish et al., 2017).

Despite the fact that asphyxia had a devastating effect on neonatal mortality and morbidity, competency in dealing with resuscitation had remained a significant challenge. (Debelew et al., 2014; Khalid et al., 2015). Many neonates in developing countries die unnecessarily from asphyxia because healthcare providers have not had the necessary knowledge and skill on how to give simple resuscitation. (Msemo et al., 2013).

Nurses and midwives had a considerable knowledge and skill gap in all areas of resuscitations (Carlo et al., 2010; World Health Organization, 2016).

Poor knowledge in diagnosing asphyxia and poor skill in neonatal resuscitation had persisted, contributing to major gaps for the quality of services.

This suggested that lack of competency in neonatal resuscitation is one of the impediments for saving the asphyxiated baby. Therefore, further research is needed to investigate barriers contributing to poor resuscitation.

Knowledge and skill fall‐off as well as poor retention of knowledge and skill shortly after training had contributed significantly towards poor resuscitation (Nour et al., 2012; Wall et al., 2009). This could be due to lack of continuous and regular supportive supervision, refreshment training or it may be associated with lack of resuscitation guidelines, equipment and supplies. Thus, continuous and regular research is needed to explore factors contributing to poor knowledge and practice and to implement strategic interventions for the improvement of the quality of newborn health care.

Despite the adverse effects of asphyxia on neonatal mortality and morbidity, poor resuscitation had persisted significantly. Barriers contributing to poor resuscitation had remained unidentified. Researches on the area of competency had remained scarce especially in low‐resource countries including Ethiopia (Khoury et al., 1985; Wolde et al., 2019). Moreover, to the best of the investigator's knowledge, there have been no similar studies done in south Wollo Zonal health institutions. Therefore, the aim of this study was to determine knowledge and skill gaps in neonatal resuscitation among nurses and midwives in south Wollo governmental hospitals. In particular, the study aims to investigate the lack of information regarding the impact of the on‐going teaching programme (NRP) regarding nurses in Sudan.

### 2.2 Justification

This study is conducted due to the lack of information regarding the impact of this teaching programme (NRP) regarding nurses in Sudan. In addition, it also seeks to investigate the high incidence of neonatal death in the targeted area and effects of the lack of facilities in the localities.

Since 2.6 million neonates die every year due to asphyxia, midwives and nurses are expected to have appropriate knowledge and skill towards neonatal resuscitation. Given the serious threat of asphyxia, HCPs must be aware on how to provide successful neonatal resuscitations. Continuously boosting the knowledge and practice of HCPs regarding neonatal resuscitation is very crucial because knowledge and practice change over time as new evidence arises. Therefore, it is important to conduct regular and continuous assessment of skills and knowledge to keep them congruent with the current knowledge and practice. The policymakers, hospital managers and other stakeholders must pay due attention to planning, implementing and evaluating various interventions related to neonatal morbidity and mortality.

## 3 THE STUDY

### 3.1 Design

This study is a quasi‐experimental quantitative study conducted in Sudan, White Nile state, to evaluate the effectiveness of designed guidelines regarding advanced neonatal resuscitation for nurses and midwives during the period starting from August 2019–January 2021. (and also has been guided by STROBE, cohort study) checklist (see File S1).

#### 3.1.1 Study area/setting

The study was conducted at White Nile State, Kosti and Rabak locality, which is located in the west of Khartoum state. The area of the locality is about 303 kilometres from the centre. White Nile State is located in the southern part of the country. The state has an area of 39,701 square kilometres. It is bordered to the north by Khartoum State, to the west by North Kordofan State, to the southwest by South Kordofan State, by Upper Nile State to the south, and to the east by the states of Gezira and Sennar. The state population is about 1,726,356 people, according to 2002 census.

### 3.2 Method

In this study, I use the quasi‐experimental, quantitative approach to measure the effect of the training programme on nurses' knowledge, practice and subsequences neonatal mortality rate. Data were collected by using two tools: semi‐structured questionnaire and checklist.

First pre‐test was conducted for participants to measure their knowledge, and then training programme has been conducted with the following objectives in mind: (1) to increase the awareness of the staff regarding the (NRP) programme and (2) to improve the skills and practice regarding (NRP) programme and decrease neonatal mortality rate. Nurses and midwives were divided into groups according to their working hours and working place. Materials which are used for the training programme included brochures and printout pictures. In addition, theory and practice sessions have been held (simulation). A posttest was provided 3 months after conducting the education programme, and neonatal mortality rate has been checked. Pre‐ and post‐questionnaire are same, and a checklist was used for measuring the practice.

In the evaluation, the practice scenario was conducted for the participant and recorded with video and then checked for errors. Unwanted videos were then deleted.

During the training programme, handouts were distributed, and at the end of the training activities, policies and guidelines were provided, and selection of the best participants done to engage in (TOT) Training of Trainers programme. Follow‐up was carried out 4 months later and postquestionnaire and checklist applied to the participants.

### 3.3 Analysis

Prior to analysis, data were prepared and arranged in excel sheets and then entered in statistical analyses programme called SPSS (version 22; SPSS Inc., Chicago, IL) software. Data were expressed as percentages. Descriptive analyses of percentages of categorical variables were reported using Chi‐squared test (*χ* ^2^). An alpha value of <0.05 denoted a statistically significant difference in all statistical comprises. The paired sample t test has been used so as to test the difference between pre‐ and post‐scoring after and before intervention.

### 3.4 Ethics

Permission was taken from Bahri University and from the administration of the Ministry of Health in the White Nile State. In addition, permission has also been taken from Kosti hospital and Rabak hospital where the study has been conducted. The researcher introduced herself to the nurses and midwives who met the inclusion criteria. They were informed that they could either participate or decline participation and are free to discontinue participation at any time as they wish. The researcher obtained verbal consent from each participant. Confidentiality was maintained by ensuring that respondents' names are not endorsed on the questionnaire forms. This was done to safeguard the rights of the respondents to be free to answer all questions.

## 4 RESULTS

**FIGURE 1:**
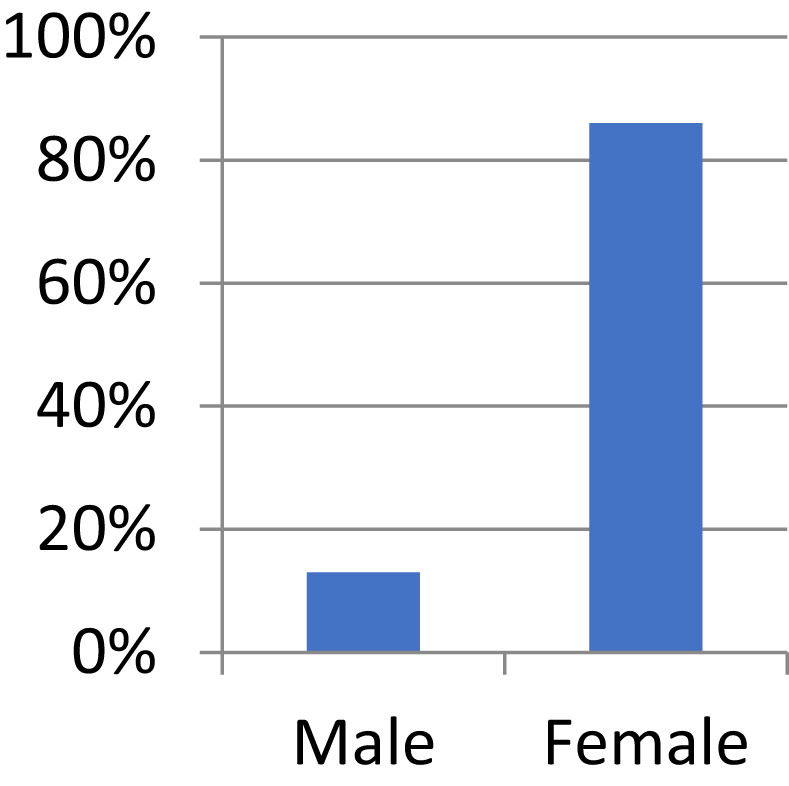
Distribution of respondents according to gender (*N* = 75)

**FIGURE 2:**
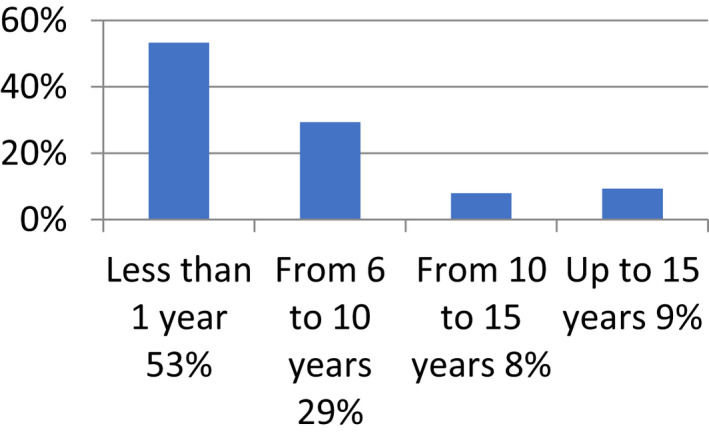
Distribution of respondents according to years of experience in neonatal resuscitation

**FIGURE 3:**
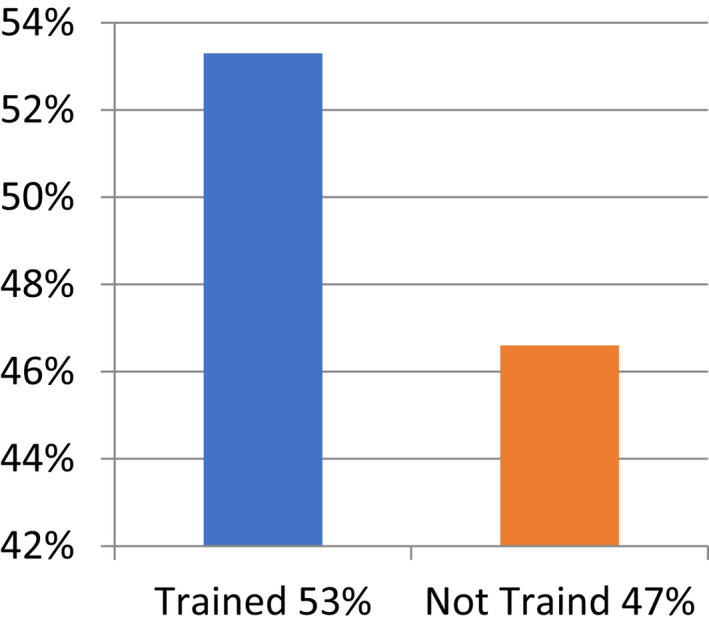
5/Distribution of respondents according to receiving training regarding neonatal resuscitation

**FIGURE 4:**
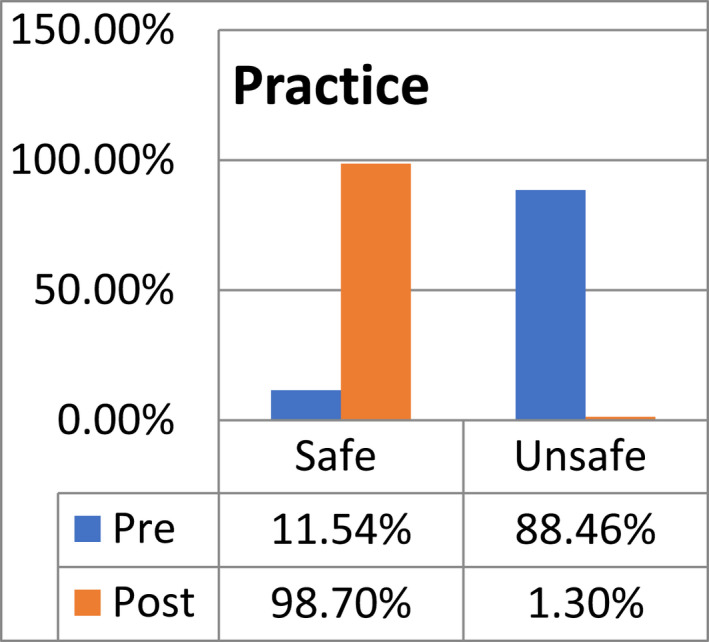
Distribution of practice among Rabak&Kosti Hospitals workers (*N* = 75)

The level of significance was set at *p* < .05 (Figures 1, 2, 3, 4 and Table 1).

**TABLE 1:** Association between knowledge and professional skill among Rabak & Kosti Hospitals workers: (*N* = 75)

Field of study/profession	(pre)		(post)	
	Frequency	Percentage	Frequency	Percentage
General nursing	25	37.8%	37	56.0%
Midwifery	1	12.5%	2	25.0%
Total	75	100.0	75	100.0

*Note*: The table shows that the most knowledgeable respondents are from nurses.

T* *p*‐value <.05 significantly different.

## 5 DISCUSSION

This quasi‐experimental study was conducted to study the effect of educational programme of health workers (nurses and midwifes) related to advanced neonatal resuscitation in Kosti and Rabak hospital's Neonatal Intensive Care Unit, Obstetric Emergency Rooms, Delivery rooms, Obstetric Operation rooms, postnatal wards and paediatric and neonatal wards.

A total of 72% of the respondents had a Diploma degree, 20% had a Bachelor, 6.7% had a Master and 1.3% were Ph.D. holders. The research findings agree with previous studies in India and Afghanistan (2013) which found no significant difference in skill and knowledge as indicated by academic qualifications. Likewise, a study in Nigeria (2016) had suggested that years of service and educational level had no significant relationship with nurses' level of knowledge in the management of birth asphyxia, but nurses with paediatric specializations showed significant positive association with knowledge of neonatal resuscitation. (Ashish et al., 2017).

The years of experience in neonatal resuscitation of respondents is 53.3% (<1 year), According to the research findings, this has strongly affected the performance of the respondents which is also in line with other studies in Kenya and Afghanistan which showed that providers' years of experience was positively associated with performance of neonatal resuscitation. (Kim et al., 2013; Shikuko et al., 2017; Wall et al., 2009; Wolde et al., 2019).

The training statistics indicate that (53.3%) of the respondents received training but (46.7) % of the respondents did not although they got basic neonatal CPR. The present research findings indicate that training has a direct affect upon performance with respect to basic neonatal CPR.

As for newborn resuscitation policies, 74.7% of the respondents indicated that Kosti and Rabak Hospitals had no policies and instructions for performing resuscitation for newborns, while 76% of the respondents stated that the hospitals do not provide them with adequate supervisory support for newborn resuscitation. These findings agree with those of previous studies which have stated that institutional factors do contribute to the health setup which may affect providers' performance of neonatal resuscitation and consequently neonatal outcomes.

The total post‐educational programme knowledge of the respondents regarding the advanced newborn cardiopulmonary resuscitation were 49 (65.3) % compared with the pre‐educational programme knowledge, which indicates high motivation to acquire knowledge. Along similar lines, other studies in India, Nepal, Afghanistan, Tanzania and Kenya had suggested that, other things remaining equal, training had a significant effect on providers, and that there are significant differences between pre‐ and post‐intervention through the educational programme. By contrast, other studies in Baghdad and Nigeria had indicated that there was no statistically significant association between nurses' practices and their number of training sessions. (Khalid et al., 2015; Nour et al., 2012).

The total safe practice of respondents regarding protecting the patients from the risk related to newborn cardiopulmonary resuscitation were 74 (98.3)% compared with the pre‐educational programme knowledge, which clearly indicates an increase in safe practice and good measures. Likewise, a meta‐analysis of three facility‐based studies (India, Bulgaria and Zambia) suggests that training was associated with a 30% reduction in intrapartum‐related mortality and 38% reduction in early neonatal mortality. (Msemo et al., 2013).

Turning to correlation between knowledge and practice regarding advanced newborn cardiopulmonary resuscitation among workers in Kosti and Rabak hospitals, the Pearson correlation factor were found to be 0.3 and the *p*‐value is 0.0.

The respondents who had the highest level of knowledge at Kosti and Rabak hospitals were specialized in general nursing (37 [56%]), which indicates a high motivation to acquire knowledge related to advanced newborn cardiopulmonary resuscitation among nursing science certificate holder compared with midwifery certificate holder.

### 5.1 Conclusion

Based on the findings, the study concluded that the respondents' body of knowledge clearly increased after the intervention and with a consequent improvement in their performance.

### 5.2 Recommendation

Based on the research conclusions, the researcher recommends the following for White Nile State Ministry of Health, Kosti and Rabak Maternity and child health hospital:

- Continued implementation of education programmes to enhance knowledge and practice in advanced neonatal resuscitation for nurses and midwives.
- Careful preparation and implementation of policies and procedures related to neonatal care and resuscitation.
- Enhance availability of tools and equipment for neonatal care and resuscitation to save babies' lives.
- Continued supervision and monitoring of the quality of the neonatal resuscitation services.

## 6 LIMITATIONS OF THE STUDY

The study needs to cover more population, and also the educational intervention effect is not long‐term, but it was mentioned in the study re‐recommendation to do continuing education to ensure the quality of neonatal care services.

## 7 CONCLUSION

Based on its own findings, the study concluded that the respondents' body of knowledge increased considerably after the intervention, their performance was improved and neonatal mortality rate has been decreased. The study recommends that continuous educational programmes be carried out, policies and procedures related to neonatal care and resuscitation should be carefully prepared and implemented, tools and equipment for neonatal care and resuscitation must be made available to save babies' lives, providing needed training to the relevant physicians in advanced neonatal resuscitation as integral part of their qualification, supervising and continuously monitoring the quality of the services provided.

## Supporting information

File S1
